# Positive correlations between TyG and TyG-BMI indices and the risk of NAFLD and degree of liver fibrosis in patients undergoing PCI

**DOI:** 10.3389/fendo.2025.1541421

**Published:** 2025-09-29

**Authors:** Yingxiang Chen, Che Wang, Xiaoyu Du, Xiaotong Sun, Wenjuan Song, Chengzhi Lu

**Affiliations:** ^1^ School of Medicine, Nankai University, Tianjin, China; ^2^ Clinical Medical College of Tianjin Medical University, Tianjin, China; ^3^ Department of Cardiology, Tianjin First Central Hospital, Tianjin, China

**Keywords:** non-alcoholic fatty liver disease, PCI, TyG, TyG-BMI, insulin resistance

## Abstract

**Background:**

We aim to investigate the association between TyG(Triglyceride-Glucose index) and TyG-BMI(Triglyceride-Glucose-Body Mass Index) indices and the risk of non-alcoholic fatty liver disease (NAFLD) in patients undergoing percutaneous coronary intervention (PCI), an area where their predictive value is currently unclear, despite their established link to insulin resistance, metabolic syndrome, and cardiovascular disease.

**Methods:**

In this cross-sectional study, 776 patients who underwent coronary angiography and PCI were categorized into NAFLD+PCI and PCI groups based on abdominal ultrasound. They were further classified by TyG and TyG-BMI indices. Continuous variables were compared using ANOVA, Wilcoxon-Mann-Whitney, or t-tests, while categorical variables were analyzed with χ² or Fisher exact tests. Logistic regression identified independent factors for NAFLD in PCI patients. ROC curves evaluated the predictive efficacy of TyG and TyG-BMI for NAFLD. Linear correlation and multiple linear regression assessed relationships among NAFLD fibrosis score (NFS), TyG, and TyG-BMI.

**Results:**

Among 776 patients, NAFLD was detected in 305. After adjusting for age, smoking, hypertension, diabetes, sex, and cardiovascular disease, multivariate logistic regression showed the TyG index was a significant risk factor for NAFLD in PCI patients (OR = 2.04; 95% CI, 1.62-2.55; P < 0.001). Similarly, the TyG-BMI index, total cholesterol, triglycerides, LDL cholesterol, fasting blood glucose, and BMI were associated with increased NAFLD risk. Each unit increase in the TyG index raised the NAFLD risk by 2.63-fold (OR = 2.63; 95% CI, 1.78-3.8; P<0.001), and each unit increase in the TyG-BMI index by 3.80-fold (OR = 3.80; 95% CI, 2.55-5.68; P < 0.001). Multivariate linear regression indicated that in the PCI-NAFLD group, each unit increase in the TyG index increased the NFS value by 0.247 (β = 0.247; 95% CI, 0.19-0.45; P < 0.001), and each unit increase in the TyG-BMI index increased the NFS value by 0.344 (β = 0.344; 95% CI, 0.28-0.59; P < 0.001).

**Conclusions:**

The TyG index and TyG-BMI were positively associated with the risk of NAFLD in patients treated with PCI, reflecting the severity of liver fibrosis.

## 1 Introduction

Non-alcoholic fatty liver disease (NAFLD), a liver disease unrelated to alcohol consumption and characterized by an abnormal accumulation of fat in liver tissue, is a significant public health challenge worldwide (1). Current estimates indicate that the prevalence of NAFLD in adults approximately 20–30%; in certain populations, such as overweight or obese individuals and patients with type 2 diabetes, this proportion increases significantly and can reach 50—75%. The developmental mechanisms of NAFLD are extremely complex and include genetic predisposition, insulin resistance, chronic mild inflammatory responses and endoplasmic reticulum stress (2–4). Recent studies show a positive correlation between the TyG index and NAFLD severity. The TyG index, reflecting insulin sensitivity through triglyceride and fasting blood glucose levels, enhances our understanding of NAFLD’s pathophysiology and may provide new approaches for early detection and intervention (5–8).

Cardiovascular disease (CVD) is the leading global cause of mortality. Percutaneous coronary intervention (PCI) effectively treats coronary artery narrowing or blockage, reducing mortality and rehospitalization in acute coronary syndrome patients. Patients undergoing PCI often have metabolic disorders such as hypertension, elevated blood sugar, and lipid metabolism issues, which are closely linked to NAFLD development and progression (9, 10).

The triglyceride-glucose index (TyG), which combines fasting glucose and triglyceride levels, is a marker for insulin resistance (IR). The TyG-BMI extends this by incorporating body mass index (BMI), further clarifying the link between IR and overweight. Recent studies show that the TyG index is significantly associated with cardiovascular risks such as hypertension, hyperlipidemia, and type 2 diabetes (11–13). However, few studies have examined the predictive ability of IR for NAFLD risk in PCI patients. Thus, this study focuses on high-risk PCI patients to analyze the relationships between the TyG index and TyG-BMI and the risk of NAFLD. This is crucial for identifying high-risk patients, developing preventive strategies, and improving patient outcomes.

## 2 Data and methods

### 2.1 Subjects and study design

**Figure 1:**
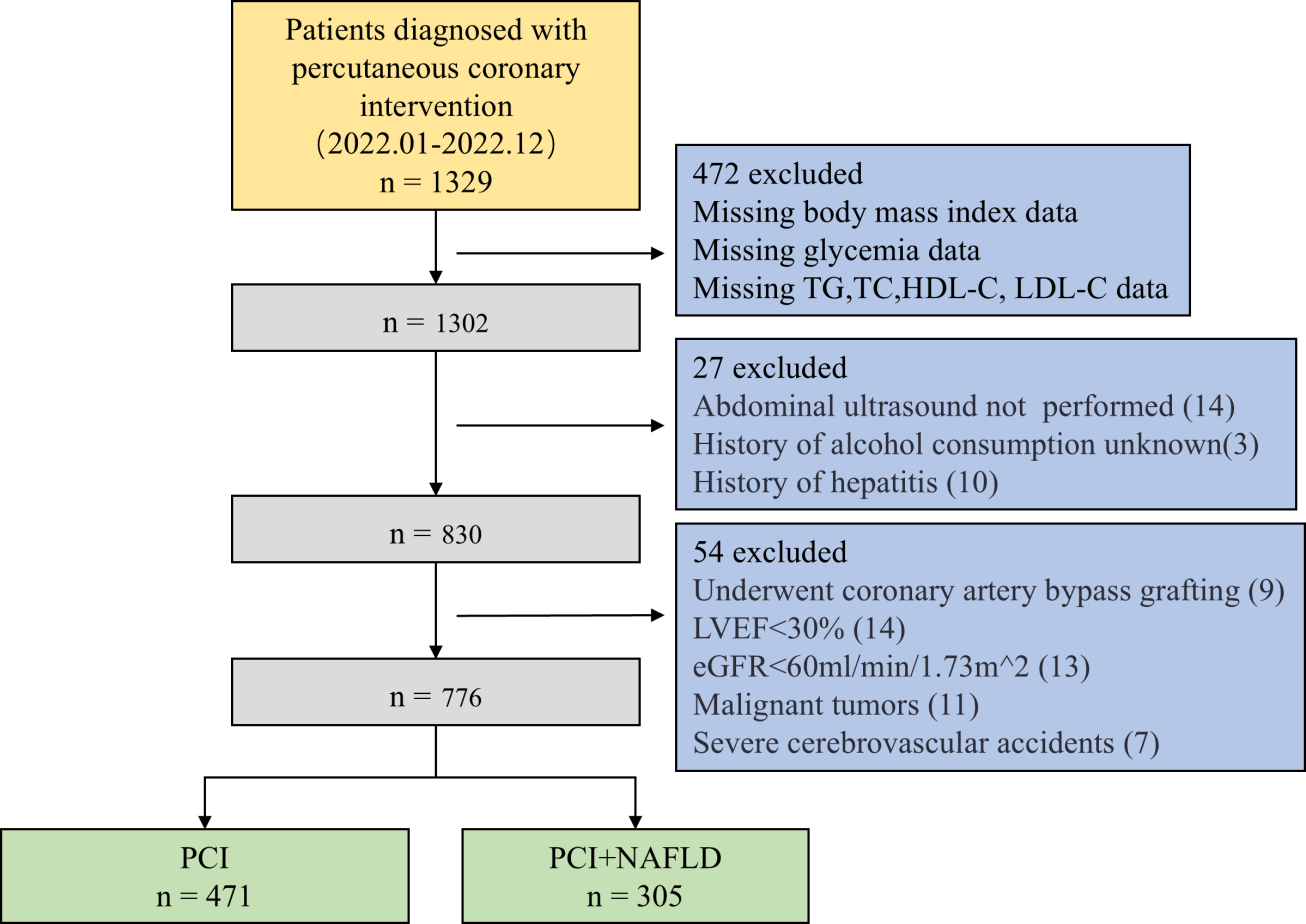
Subject selection flowchart. TG, triglycerides; TC, total cholesterol; HDL-C, high-density lipoprotein cholesterol; LDL-C, low-density lipoprotein cholesterol; LVEF, left ventricular ejection fraction; eGFR, estimated glomerular filtration rate.

This cross-sectional study included 1329 patients hospitalized in the Department of Cardiology of Tianjin First Central Hospital between January and December 2022, who underwent coronary angiography and PCI. As shown in **Figure 1**, after excluding patients with key data loss and confounding data, 776 patients remained. The patients were divided into two groups based on abdominal ultrasonography results: NAFLD+PCI and PCI groups.

The NAFLD Fibrosis Score (NFS) is a mathematical model constructed from six clinical parameters—age, body mass index (BMI), hyperglycemia (impaired fasting glucose), platelet count, albumin level, and the AST/ALT ratio—designed for the noninvasive quantification of liver fibrosis severity in patients with nonalcoholic fatty liver disease (NAFLD). An NFS > 0.676 indicates significant fibrosis, while an NFS < -1.455 suggests advanced liver fibrosis.

The NFS score was calculated via the formula: NFS = -1.675 + 0.037 × age (years) + 0.094 × BMI (kg/m²) + 1.13 × TDM (yes = 1, no = 0) + 0.99 × AST/ALT - 0.013 × plt (×10^9/L) - 0.66 × Alb (g/dL).

NAFLD diagnostic criteria include: alcohol consumption <140 g/week for men and <70 g/week for women, exclusion of other chronic liver diseases (e.g., hepatitis B/C, autoimmune conditions, primary biliary cirrhosis, haemochromatosis, Wilson’s disease), and ultrasound confirmation of hepatic steatosis.

Patients with a history of coronary artery bypass grafting (CABG), other heart conditions, malignant tumors, autoimmune diseases, infections, severe cerebrovascular events, or severe kidney disease were excluded.

### 2.2 Data collection and measurements

Demographic characteristics, test results, and laboratory values were collected from electronic medical records. This included sex, age, height, weight, smoking and alcohol history, admission rhythm, and clinical information such as hypertension, diabetes, and cardiovascular disease.

The following morning after overnight fasting, a 3 mL venous blood sample was drawn from the antecubital fossa and placed in an anticoagulation tube. An enzyme-linked immunosorbent assay (ELISA) was used to measure hematological parameters (white blood cell (WBC) count, red blood cell (RBC) count, hemoglobin, and platelet count) and biochemical indicators (alanine transaminase (ALT), aspartate aminotransferase (AST), serum creatinine (Scr), total cholesterol (TC), triglycerides (TG), high-density lipoprotein cholesterol (HDL-C), low-density lipoprotein cholesterol (LDL-C), fasting blood glucose (FBG), C-reactive protein (CRP), and B-type natriuretic peptide (BNP)).

The body mass index (BMI), TyG index, and TyG-BMI index were calculated. BMI was calculated as weight (kg)/height² (m²). The TyG index was calculated using the formula: Ln[TG (mg/dL) × FBG (mg/dL)/2]. The TyG-BMI index was derived by multiplying the TyG index by the BMI.

### 2.3 Statistical analysis

Data were analyzed using SPSS 26.0, GraphPad Prism 8.0, and R 4.3.3. The Shapiro-Wilk test assessed the normal distribution of continuous variables. Normally distributed data are presented as mean ± standard deviation (x̅ ± s) and analyzed using the independent samples t-test. Non-normally distributed data are presented as median [interquartile range (Q1, Q3)] and analyzed using the Wilcoxon-Mann-Whitney test. Frequency data are presented as n (%) and analyzed using the chi-square test (χ²) or Fisher’s exact test. A p-value < 0.05 indicates statistical significance.

Univariate logistic regression was performed to identify potential predictive factors for NAFLD, followed by multivariate logistic regression to determine independent predictors and their effectiveness. The baseline characteristics of the TyG and TyG-BMI groups were compared using one-way ANOVA for continuous variables and the Kruskal-Wallis test for nonparametric data. Categorical variables were compared using the chi-square (χ²) or Fisher’s exact test. Univariate and multivariate logistic regressions assessed the impact of TyG and TyG-BMI levels on NAFLD risk. ROC curve analysis evaluated the predictive performance of TyG and TyG-BMI indices, based on AUC values. Linear correlation and multivariate linear regression were used to assess associations between the NFS score and the TyG and TyG-BMI indices.

## 3 Results

### 3.1 Demographic and clinical characteristics of patients who underwent PCI with and without NAFLD

Data were obtained from 776 PCI patients (516 men, 260 women), including 305 with NAFLD and 471 without. The median ages (IQR) were 67 years (60-73) for the PCI group and 65 years (56-71) for the PCI plus NAFLD group. **Table 1** shows the demographic and clinical characteristics of both groups. There were no significant differences in admission rhythm, smoking status, diabetes prevalence, cardiovascular disease incidence, ALT, AST, Scr, eGFR, WBC, RBC, PLT, hs-CRP, BNP, and HDL-C. However, the PCI plus NAFLD group had a lower proportion of males but a higher proportion with hypertension, a lower median age, and higher mean BMI, TC, TG, LDL-C, FBG, TyG index, and TyG-BMI.

**Table 1:** Demographic and clinical characteristics of participants by the presence of PCI.

Variables	PCI(n=471)	PCI+NAFLD(n=305)	SUM(n=776)	*t/X^2^/Z*	*P*
Sex(male)	316(67.09%)	203(66.56%)	519(66.88%)	0.024	0.877
Age(years)	67(60,73)	65(56,71)	66(59,72))	2.307	0.021
BMI(kg/m^2^)	24.93(22.88,27.27)	26.57 (24.49,28.76)	25.69 (23.44,27.89)	-6.301	<0.001
HR(bpm)	72(65,80.5)	74(65,82)	72(65,81)	-1.178	0.239
Smoke(n%)	212(45.01%)	127(41.64%)	339(43.69%)	0.855	0.355
HT(n%)	315(66.88%)	225(73.77%)	540(69.59%)	4.154	0.042
DM(n%)	177(37.58%)	120(39.34%)	297(38.27%)	0.244	0.621
CVD(n%)	94(19.96%)	57(18.69%)	151(19.46%)	0.190	0.663
ALT(U/L)	18.5(13.1,25.05)	19.2(13.1,28.4)	8.8(13.1,26.92)	-1.732	0.083
AST(U/L)	18.4(15,25.3)	18.6(14.7,23.3)	18.4(14.9,24)	0.550	0.583
Scr(mmol/L)	75.4(64,85)	74(63,89)	75(63,87.05)	0.053	0.958
eGFR(mL/(min/1.73m^2^))	87.28(73.47,95.61)	89.07(74.65,98.58)	87.55(74.09,96.55)	-1.732	0.083
WBC(*10^9^/L)	6.61(5.46,7.95)	6.98(5.66,8.25)	6.74(5.52,8.09)	-1.758	0.079
RBC(*10^12^/L)	4.4(4.13,4.87)	4.54(4.21,4.93)	4.5(4.17,4.89)	-1.527	0.127
PLT(*10^9^/L)	213(178.5,252.5)	222(190,259)	217(182,256)	-2.129	0.033
hs-CRP(mg/L)	1.34(0.54,4.1)	1.85(0.77,4.41)	1.54(0.64,4.27)	-2.174	0.03
BNP(pg/Ml)	178.4(69.47,611.8)	136.9(56.63,412.9)	160.8(64.67,515.98)	2.248	0.025
TC(mmol/L)	3.96(3.24,4.77)	4.44(3.69,5.44)	4.14(3.37,5.07)	-5.494	<0.001
TG(mmol/L)	1.34(0.97,1.98)	1.77(1.28,2.59)	1.48(1.03,2.19)	-6.646	<0.001
HDL-C(mmol/L)	1.04(0.9,1.24)	1.01(0.89,1.2)	1.03(0.9,1.22)	1.383	0.167
LDL-C(mmol/L)	2.49(1.87,3.22)	2.77(2.16,3.78)	2.63(1.96,3.46)	-4.521	<0.001
FBG(mmol/L)	6.22(5.24,8.18)	6.58(5.32,9.01)	6.36(5.27,8.48)	2.022	0.043
TyG	1.46(1.04,1.97)	1.81(1.36,2.31)	1.6(1.16,2.12)	-6.373	<0.001
TyG-BMI	36.51(25.73,50.43)	48(34.98,64.5)	40.68(28.98,56.06)	-7.279	<0.001

### 3.2 Univariate and multivariate analysis of factors associated with NAFLD in patients undergoing PCI

**Figure 2:**
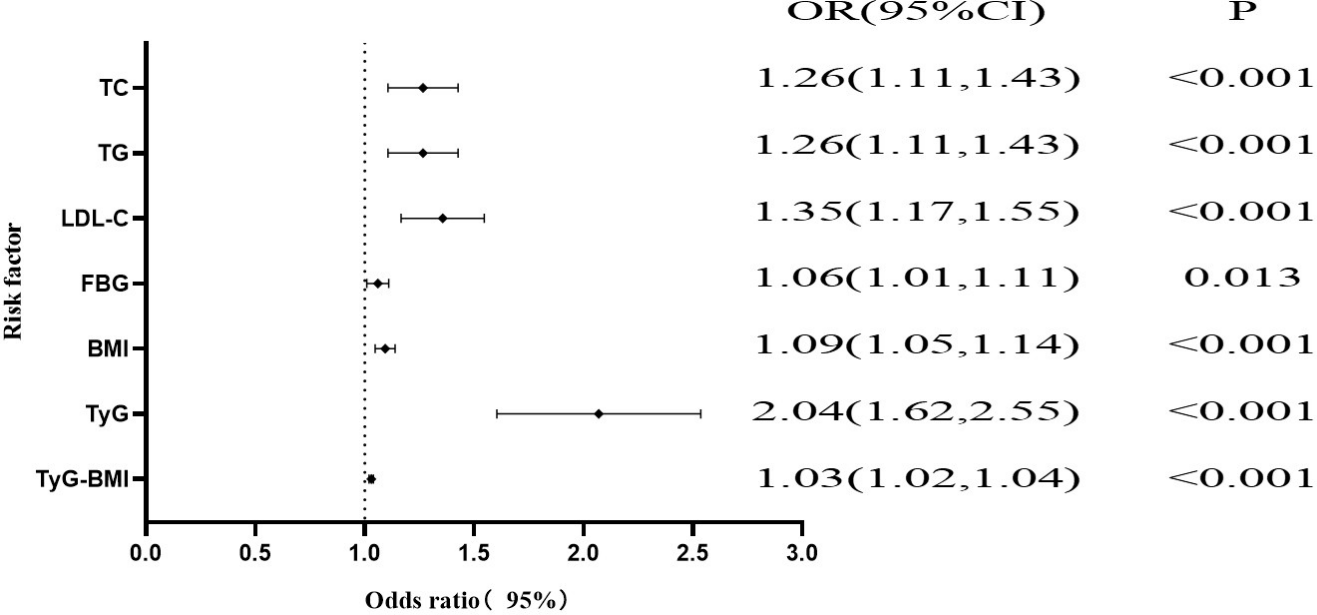
Forest plots of independent factors associated with CHD in NAFLD patients.

As shown in **Figure 2**; **Table 2**, univariate logistic regression revealed associations between PLT, TC, TG, LDL-C, FBG, BMI, TyG, and TyG-BMI and NAFLD in PCI patients. Multivariate logistic regression, adjusting for other variables, showed that PLT was not a risk factor for NAFLD. TC (OR, 1.26; 95% CI, 1.11-1.43; P < 0.001), TG (OR, 1.26; 95% CI, 1.11-1.43; P < 0.001), LDL-C (OR, 1.35; 95% CI, 1.17-1.55; P < 0.001), FBG (OR, 1.06; 95% CI, 1.01-1.11; P = 0.013), BMI (OR, 1.09; 95% CI, 1.05-1.14; P < 0.001), TyG (OR, 2.04; 95% CI, 1.62-2.55; P < 0.001), and TyG-BMI (OR, 1.03; 95% CI, 1.02-1.04; P < 0.001) remained significant risk factors for NAFLD.

**Table 2:** Univariate and multivariate analyzeds of factors associated with NAFLD in PCI.

Variables	Non-adjusted		Model I		Model II	
	OR(95%CI)	*P*	OR(95%CI)	*P*	OR(95%CI)	*P*
HR(bpm)	1.01(0.99,1.02)	0.348				
ALT(U/L)	1.01(1,1.02)	0.076				
AST(U/L)	1(1,1.01)	0.618				
Scr(mmol/L)	1(0.99,1)	0.103				
eGFR(mL/(min/1.73m^2^))	1.01(1,1.01)	0.084				
WBC(*10^9^/L)	1.06(0.99,1.13)	0.091				
RBC(*10^12^/L)	1.2(0.94,1.53)	0.147				
PLT(*10^9^/L)	1(1,1.01)	0.013	1(1,1)	0.065		
hs-CRP(mg/L)	1(0.99,1.01)	0.473				
BNP(pg/Ml)	1(1,1)	0.079				
TC(mmol/L)	1.26(1.12,1.43)	<0.001	1.26(1.11,1.43)	<0.001	1.26(1.11,1.43)	<0.001
TG(mmol/L)	1.51(1.32,1.73)	<0.001	1.48(1.29,1.7)	<0.001	1.26(1.11,1.43)	<0.001
HDL-C(mmol/L)	0.71(0.42,1.19)	0.19				
LDL-C(mmol/L)	1.34(1.17,1.54)	<0.001	1.34(1.17,1.54)	<0.001	1.35(1.17,1.55)	<0.001
FBG(mmol/L)	1.05(1.01,1.1)	0.016	1.05(1.01,1.1)	0.016	1.06(1.01,1.11)	0.013
BMI(kg/m^2^)	1.11(1.06,1.15)	<0.001	1.1(1.06,1.15)	<0.001	1.09(1.05,1.14)	<0.001
TyG	2.01(1.63,2.48)	<0.001	1.96(1.58,2.42)	<0.001	2.04(1.62,2.55)	<0.001
TyG-BMI	1.03(1.02,1.03)	<0.001	1.03(1.02,1.03)	<0.001	1.03(1.02,1.04)	<0.001

None, non-adjusted model. Model I was adjusted for age and sex. Model II was adjusted for age, sex, hypertension, diabetes mellitus, cardiovascular disease and smoking history.

### 3.3 Demographic and clinical characteristics associated with the TyG index

To investigate the associations between different levels of insulin resistance (according to the TyG index) and NAFLD in patients undergoing PCI, patients were categorized into three groups according to TyG index tertiles: Group 1 (n = 259) with TyG index ≤ 2.00; Group 2 (n = 259) with a 2.00 < TyG index ≤ 2.61; and Group 3 (n = 258) with a TyG index > 2.61. The mean TyG indices of these groups were 1.67 (1.47, 1.85), 2.30 (2.15, 2.46), and 3.03 (2.82, 3.47), respectively. As shown in **Table 3**, the statistical analysis revealed significant differences among the three groups in terms of the incidence of NAFLD, diabetes mellitus (DM), ALT, Scr, eGFR, WBC, RBC, PLT, hs-CRP, BNP, BMI, TC, TG, HDL-C, LDL-C, FBG, TyG index and TyG-BMI index (all P values < 0.05).

**Table 3:** Demographic and clinical characteristics of participants by TyG index.

Variables	Tertile1(n=259)	Tertile2(n=259)	Tertile3(n=258)	*t/X^2^/F*	*P*
Sex(male)	178(68.73%)	168(64.86%)	173(67.05%)	0.877	0.645
Age(years)	68(60,73)	67(59,73)	64(57,69)	18.113	<0.001
BMI(kg/m^2^)	25.06(22.64,27.33)	25.61(23.58,27.78)	26.12(24.18,28.7)	20.347	<0.001
HR(bpm)	70(63,79)	72(65,80)	75(67,83)	13.889	0.001
Smoke(n%)	115(44.4%)	99(38.22%)	125(48.45%)	5.575	0.062
HT(n%)	168(64.86%)	181(69.88%)	191(74.03%)	5.147	0.076
DM(n%)	57(22.01%)	94(36.29%)	146(56.59%)	66.070	<0.001
CVD(n%)	56(21.62%)	51(19.69%)	44(17.05%)	1.734	0.42
ALT(U/L)	17.5(13.05,24.65)	17.3(12.4,24.35)	20.95(14.8,30.08)	20.875	<0.001
AST(U/L)	18.7(15.15,25)	17.5(14.65,22.9)	18.7(14.9,25.5)	3.288	0.193
Scr(mmol/L)	76(63.5,87)	78(66,90)	71.75(62,83)	11.621	0.003
eGFR(mL/(min/1.73m^2^))	86.63(74.88,94.92)	86.98(72.32,94.42)	91.32(75.87,99.56)	11.057	0.004
WBC(*10^9^/L)	6.17(5.14,7.42)	6.75(5.57,8.12)	7.22(5.94,8.6)	30.862	<0.001
RBC(*10^12^/L)	4.45(4.04,4.81)	4.5(4.24,4.84)	4.61(4.18,4.96)	7.098	0.029
PLT(*10^9^/L)	209(170,252.5)	213(182,246.5)	229.5(193.5,266)	20.958	<0.001
hs-CRP(mg/L)	1.15(0.49,3.47)	1.35(0.61,3.6)	2.28(0.97,5.1)	23.188	<0.001
BNP(pg/Ml)	218.4(72.26,625.8)	142. (62.12,416.5)	140.85(56.95,482.95)	9.149	0.01
TC(mmol/L)	3.88(3.15,4.64)	4.33(3.49,5.1)	4.38(3.7,5.42)	33.007	<0.001
TG(mmol/L)	0.94(0.74,1.12)	1.61(1.32,1.9)	2.51(1.94,3.53)	493.685	<0.001
HDL-C(mmol/L)	1.13(0.99,1.38)	1.04(0.9,1.25)	0.93(0.83,1.08)	97.475	<0.001
LDL-C(mmol/L)	2.39(1.83,3.14)	2.71(2.02,3.51)	2.72(1.99,3.62)	12.600	0.002
FBG(mmol/L)	5.47(4.91,6.35)	6.15(5.27,7.66)	9.03(6.84,12.3)	227.681	<0.001
Tyg	1.67(1.47,1.85)	2.3(2.15,2.46)	3.03(2.82,3.47)	688.047	<0.001
Tyg-BMI	41.07(33.81,48.13)	58.95(52.6,65.67)	81.81(71.87,92.54)	568.647	<0.001
NAFLD(n%)	71(27.4%)	104(40.15%)	130(50.39%)	28.715	<0.001

### 3.4 Univariate and multivariate logistic regression analysis of the TyG index groups for NAFLD

Logistic regression was performed to analyze the risk of NAFLD in the second and third groups compared with the first group as a control. After adjusting for age, sex, hypertension (HTN), DM, CVD and smoking, the results, shown in **Table 4**, indicate that a one-unit increase in the TyG index in the second group was associated with an approximately 1.79-fold increased risk of NAFLD (OR 1.79, 95% CI 1.23–2.56, P = 0.002). In the third group, a one-unit increase in the TyG index was associated with an approximately 2.63-fold increased risk of NAFLD (OR 2.63, 95% CI 1.78–3.80, P<0.001).

**Table 4:** Univariate and multivariate logistic analyzeds of NAFLD in tri-sectional TyG groups.

Group	Non-adjusted		Model I		Model II	
	OR (95%CI)	*P*	OR (95%CI)	*P*	OR (95%CI)	*P*
Tertile 1	Ref.		Ref.		Ref.	
Tertile 2	1.80(1.25,2.61)	0.002	1.78(1.23,2.58)	0.002	1.79(1.23,2.56)	0.002
Tertile 3	2.65(1.84,3.81)	<0.001	2.52(1.74,3.65)	<0.001	2.63(1.78,3.8)	<0.001

None, non-adjusted model. Model I was adjusted for age and sex. Model II was adjusted for age, sex, hypertension, diabetes mellitus, cardiovascular disease and smoking history.

### 3.5 Demographic and clinical characteristics of the TyG-BMI index

To analyzed the relationship between different levels of insulin resistance (according to the TyG-BMI index) and NAFLD in patients undergoing PCI, patients were divided into three groups according to the terciles of the TyG-BMI index: Group 1 (n = 259) with a TyG-BMI index ≤ 50.22; Group 2 (n = 259) with a 50.22 < TyG-BMI index ≤ 68.41; and Group 3 (n = 258) with a TyG-BMI index > 68.41. As shown in **Table 5**, the mean TyG-BMI indices for these groups were 40.79 (33.67, 46.11), 58.35 (54.32, 63.52), and 82.69 (74.16, 92.62), respectively. Statistical analysis revealed significant differences between the three groups in terms of the incidence of NAFLD, HTN, DM, CVD, baseline rhythm at admission, ALT, WBC, RBC, PLT, hs-CRP, BMI, TC, TG, HDL-C, LDL-C, FBG, TyG index and TyG-BMI index (all P-values < 0.05).

**Table 5:** Demographic and clinical characteristics of participants by TyG-BMI index.

Variables	Tertile1(n=259)	Tertile2(n=259)	Tertile3(n=258)	*t/X^2^/F*	*P*
Sex(male)	172 (66.41%)	175 (67.57%)	172 (66.67%)	0.086	0.958
Age(years)	68 (61,74.5)	67 (60,72)	63 (56,69)	28.110	<0.001
BMI(kg/m^2^)	23.66 (21.75,25.93)	25.42 (23.66,27.68)	27.48 (25.52,30.06)	170.115	<0.001
HR(bpm)	69 (63,78)	72 (65,80)	75 (68,83)	22.541	<0.001
Smoke(n%)	115 (44.4%)	100 (38.61%)	124 (48.06%)	4.775	0.092
HT(n%)	161 (62.16%)	184 (71.04%)	195 (75.58%)	0.003	0.958
DM(n%)	57 (22.01%)	107 (41.31%)	133 (51.55%)	49.269	<0.001
CVD(n%)	58 (22.39%)	49 (18.92%)	44 (17.05%)	2.424	<0.001
ALT(U/L)	17.5 (12.7,24.5)	17.3 (12.85,24.5)	21.45 (14.6,30.75)	22.488	<0.001
AST(U/L)	18.9 (15.15,26.45)	17.4 (14.65,22.75)	18.65 (14.8,25.5)	4.933	0.085
Scr(mmol/L)	74 (64,85.9)	78 (65,89.5)	74 (62,85)	3.903	0.142
eGFR(mL/(min/1.73m^2^))	86.5 (75.95,94.69)	87.47 (70.87,95.85)	89.33 (75.64,99.57)	4.927	0.085
WBC(*10^9^/L)	6.23 (5.14,7.38)	6.74 (5.51,7.94)	7.3 (5.94,8.68)	32.261	<0.001
RBC(*10^12^/L)	4.42 (4.04,4.8)	4.52 (4.23,4.82)	4.62 (4.21,5.02)	12.946	0.002
PLT(*10^9^/L)	211 (168.5,254)	212 (181,251)	229 (195,264)	19.554	<0.001
hs-CRP(mg/L)	1.14 (0.48,3.58)	1.25 (0.54,3.18)	2.4 (1.1,5.24)	35.097	<0.001
BNP(pg/Ml)	215 (80.77,618.1)	142.9 (57.04,426.9)	132.9 (58.15,464.82)	10.257	0.006
TC(mmol/L)	3.89 (3.15,4.64)	4.22 (3.5,5.06)	4.44 (3.69,5.44)	33.728	<0.001
TG(mmol/L)	0.97 (0.76,1.19)	1.52 (1.19,1.89)	2.44 (1.89,3.42)	438.489	<0.001
HDL-C(mmol/L)	1.17 (1,1.38)	1.03 (0.9,1.21)	0.94 (0.82,1.07)	111.749	<0.001
LDL-C(mmol/L)	2.32 (1.81,3.09)	2.74 (2,3.49)	2.72 (2.08,3.69)	17.314	<0.001
FBG(mmol/L)	5.47 (4.91,6.38)	6.35 (5.33,7.93)	8.6 (6.42,11.45)	175.840	<0.001
Tyg	1.7 (1.47,1.9)	2.3 (2.12,2.52)	3.02 (2.74,3.47)	580.959	<0.001
Tyg-BMI	40.79 (33.67,46.11)	58.35 (54.32,63.52)	82.69 (74.16,92.62)	688.889	<0.001
NAFLD(n%)	62 (23.94%)	102 (39.38%)	141 (54.65%)	51.107	<0.001

### 3.6 Univariate and multivariate logistic regression analysis of the TyG-BMI groups for NAFLD.

As shown in **Table 6**, logistic regression was performed to analyzed the risk of NAFLD in the second and third groups compared with the first group as a control. After adjusting for age, sex, HTN, DM, CVD and smoking, the results revealed that in the second group, a one-unit increase in the TyG-BMI index was associated with approximately a 2. 05-fold increased risk of NAFLD (OR 2.05, 95% CI 1.39 to 3.02, P<0.001); in the third group, a one-unit increase in the TyG-BMI index was associated with a 3.8-fold increased risk of NAFLD (OR 3.8, 95% CI 2.55 to 5.68, P<0.001).

**Table 6:** Univariate and multivariate logistic analyzeds of NAFLD in tri-sectional TyG-BMI groups.

Group	Non-adjusted		Model I		Model II	
	OR (95%CI)	*P*	OR (95%CI)	*P*	OR (95%CI)	*P*
Tertile 1	Ref.		Ref.		Ref.	
Tertile 2	2.06(1.41,3.01)	<0.001	2.04(1.39,2.98)	<0.001	2.05(1.39,3.02)	<0.001
Tertile 3	3.829(2.63,5.58)	<0.001	3.66(2.50,5.36)	<0.001	3.80(2.55,5.68)	<0.001

None, non-adjusted model. Model I was adjusted for age and sex. Model II was adjusted for age, sex, hypertension, diabetes mellitus, cardiovascular disease and smoking history.

### 3.7 ROC analysis of the TyG and TyG-BMI indices

**Figure 3:**
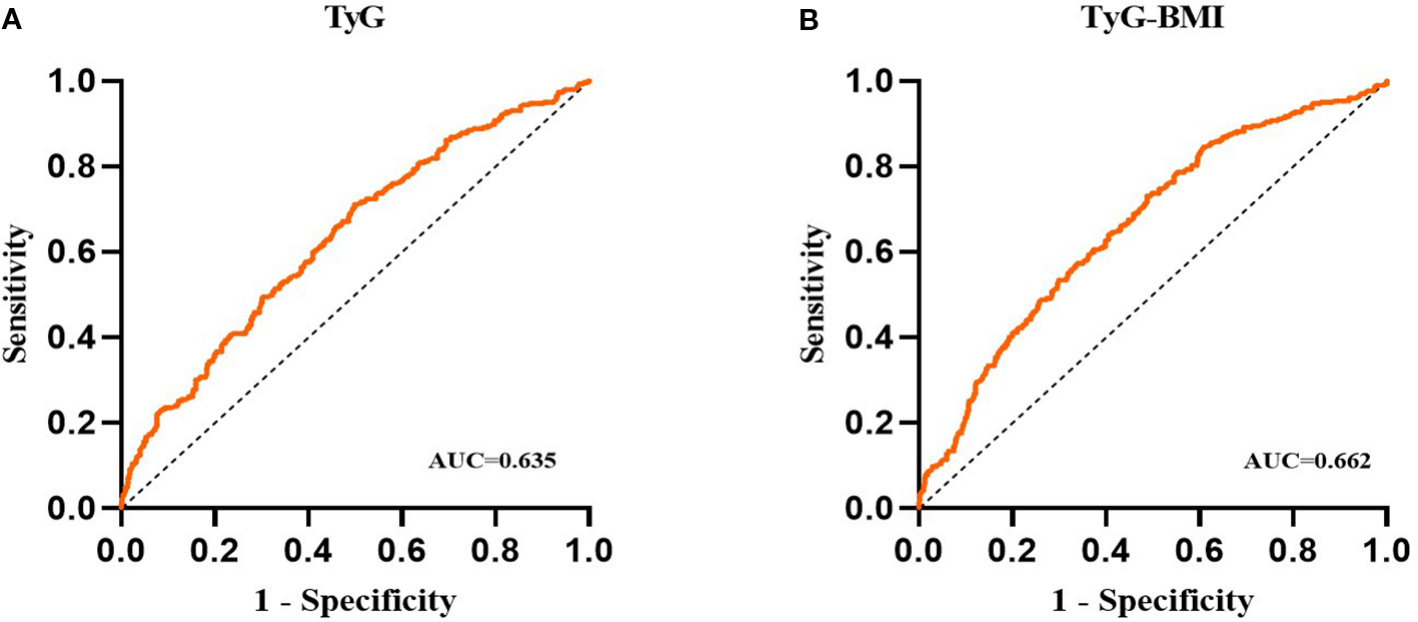
**(A)** ROC analyses of the TYG index; **(B)** ROC analyses of the TYG-BMI index.

The results of the receiver operating characteristic (ROC) analysis and visualization of the TyG and TyG-BMI indices are summarized in **Table 7**; **Figure 3**. The ROC curve for the TyG index shows an area under the curve (AUC) of 0.635 (95% CI, 0.596 to 0.675; P < 0.001), with an optimal threshold value of 2.153 for identifying NAFLD (sensitivity of 0.712, specificity of 0.501). The ROC curve for the TyG-BMI index shows an AUC of 0.662 (95% CI, 0.623 to 0.701; P < 0.001), with an optimal threshold of 54.22 to identify NAFLD (sensitivity of 0.731, specificity of 0.512). These findings suggest that both the TyG index and the TyG-BMI index have some diagnostic efficacy in predicting NAFLD.

**Table 7:** ROC curve analyzeds of TyG index and TyG-BMI index.

Index	AUC	95%CI	*P*	Sensitivity	Specificity	Optimal Cutoff
TyG	0.635	(0.596,0.675)	<0.001	0.712	0.501	>2.153
TyG-BMI	0.662	(0.623,0.701)	<0.001	0.731	0.512	>54.22

### 3.8 Associations of the TyG and TyG-BMI Indices with NAFLD Fibrosis NFS

Spearman rank correlation analysis was used to explore the relationships between NFS scores and ALT, AST, TC, TG, HDL-C, LDL-C, FBG, the TyG index, and the TyG-BMI index. As shown in **Table 8**, NFS scores did not significantly correlate with ALT (r = 0.093, P = 0.103), AST (r = -0.025, P = 0.67), TC (r = -0.018, P = 0.76), or LDL-C (r = -0.052, P = 0.362). Weak correlations were observed with TG (r = 0.216, P < 0.001) and HDL-C (r = -0.155, P = 0.007). Moderate correlations were found with FBG (r = 0.358, P < 0.001), the TyG index (r = 0.352, P < 0.001), and the TyG-BMI index (r = 0.422, P < 0.001).

**Table 8:** Relationship between NFS score with ALT AST, TG,TC, HDL-C,LDL-C, FGB, TyG and TyG-BMI.

Variables	r	*P*
ALT (U/L)	0.093	0.103
AST (U/L)	-0.025	0.67
TG (mmol/L) TC (mmol/L) HDL-C (mmol/L) LDL(mmol/L) FBG (mmol/L) Tyg Tyg-BMI	0.216 -0.018 -0.155 -0.052 0.358 0.352 0.422	<0.001 0.76 0.007 0.362 <0.001 <0.001 <0.001

**Figure 4:**
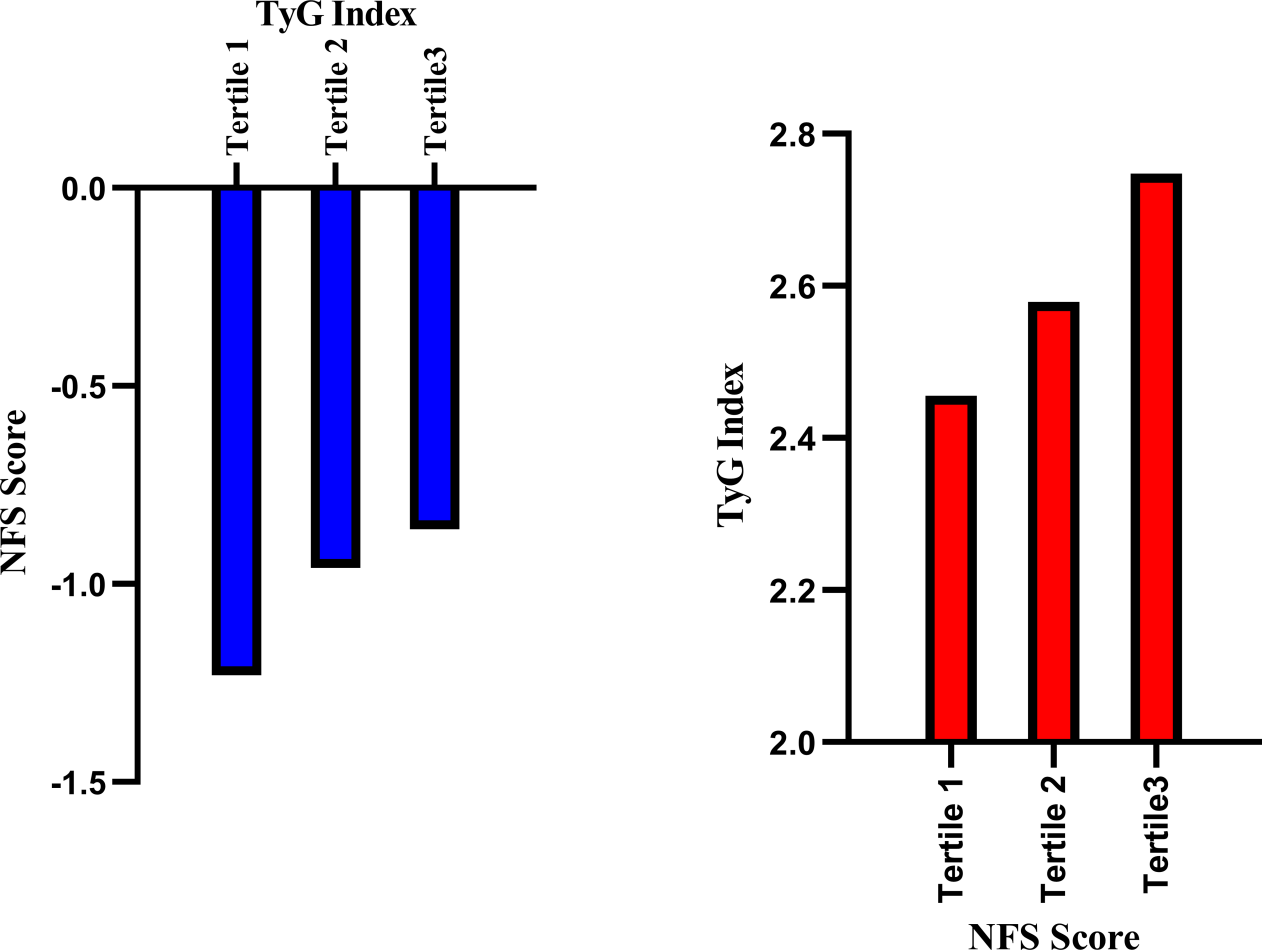
Relationships between the NFS and TyG index in PCI-NAFLD patients. The blue bar chart illustrates the NFS scores in the three TyG groups of PCI-NAFLD patients (Tertile 1 = 102, Tertile 2 = 102, Tertile 3 = 101), and the red bar chart illustrates the TyG index in the three NFS score groups of PCI-NAFLD patients (Tertile 1 = 102, Tertile 2 = 102.Tertile 3 = 101).

**Figure 5:**
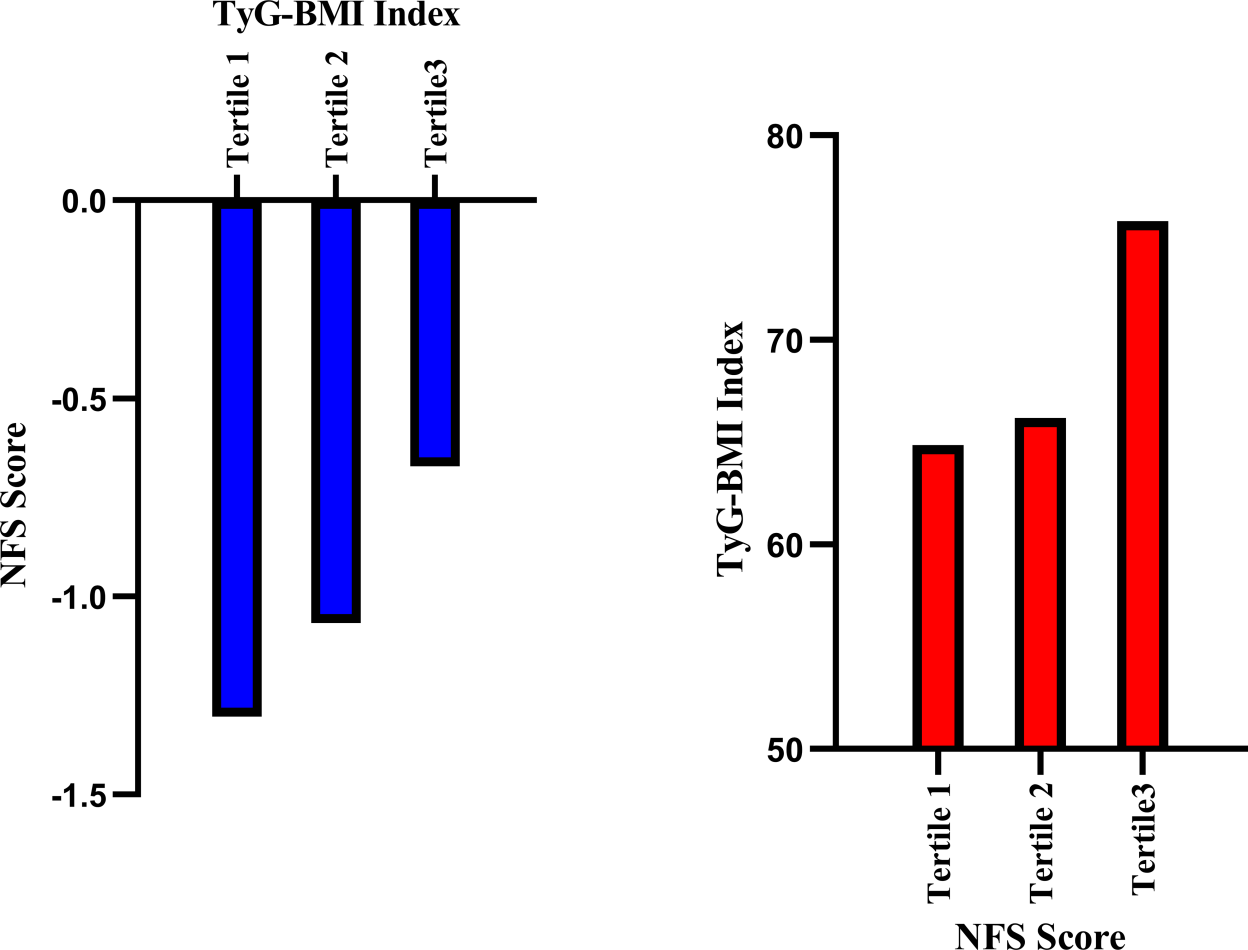
Relationships between the NFS score and the TyG-BMI in PCI-NAFLD patients. The blue bar chart illustrates the NFS score in the three-sectional TyG-BMI groups among the PCI-NAFLD patients (Tertile 1 = 102, Tertile 2 = 102, Tertile 3 = 101), and the red bar chart illustrates the TyG-BMI index in the three-sectional NFS score groups among the PCI-NAFLD patients (Tertile 1 = 102, Tertile 2 = 102, Tertile 3 = 101).

**Figures 4**, **5** respectively illustrate the NFSs of PCI patients with NAFLD grouped by TyG and TyG-BMI indices (n=102 per group), as well as the distributions of the TyG and TyG-BMI indices in PCI patients with NAFLD categorized by the NFS.

**Figure 6:**
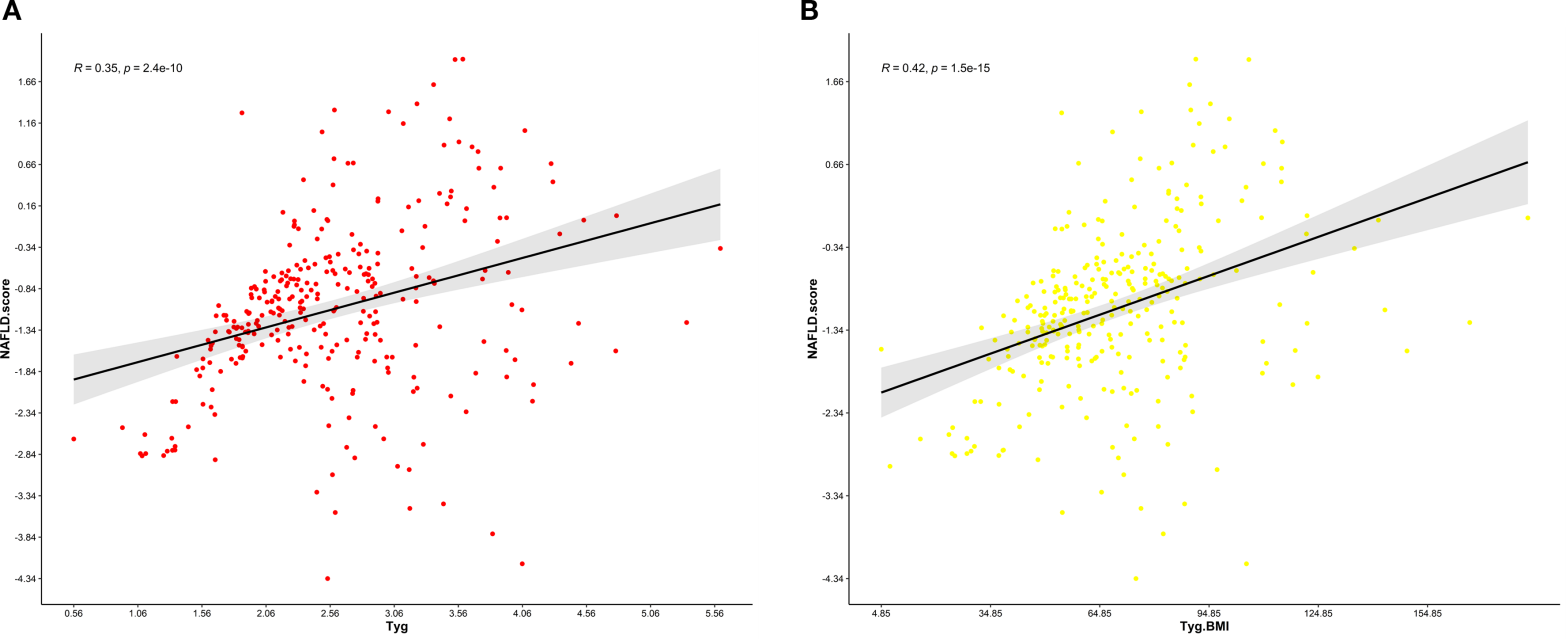
**(A)** Relationship between the NFS score and the TyG index.This scatter plot illustrates the correlation between the non-alcoholic fatty liver disease (NAFLD) fibrosis score (NFS) and the triglyceride-glucose (TyG) index in a cohort of patients. The Pearson correlation coefficient (R) is 0.35, indicating a weak positive association between the NFS and the TyG index. **(B)** Relationship between the NFS score and the TyG-BMI index. This scatter plot illustrates the correlation between the non-alcoholic fatty liver disease (NAFLD) fibrosis score (NFS) and the triglyceride-glucose-body mass index (TyG-BMI) index in a cohort of patients. The Pearson correlation coefficient (R) is 0.42, indicating a moderate positive association between the NFS and the TyG-BMI index.

**Figures 6A**, **B** present the analysis graph showing the positive correlation between NFS scores and the TyG and TyG-BMI indices. The results of the multivariate linear regression analysis are provided in **Table 9**. The analysis revealed a significant positive correlation between NFS scores and both the TyG and TyG-BMI indices. In patients with PCI and NAFLD, an increase of one unit in the TyG index corresponds to a 0.325 increase in the NFS score (β = 0.325; 95% CI, 0.28 to 0.56; P<0.001); an increase of one unit in the TyG-BMI index corresponds to a 0.376 increase in the NFS score (β = 0.376; 95% CI, 0.31 to 0.63, P<0.001).

**Table 9:** Univariate and multivariate linear regression of NFS scores.

Index	Non-adjuste			Model I			Model II		
	β	95%CI	*P*	β	95%CI	*P*	β	95%CI	*P*
TyG	0.325	(0.28,0.56)	<0.001	0.367	(0.34,0.61)	<0.001	0.247	(0.19,0.45)	<0.001
TyG-BMI	0.376	(0.31,0.63)	<0.001	0.452	(0.38,0.67)	<0.001	0.344	(0.28,0.59)	<0.001

None, non-adjusted model. Model I was adjusted for age and sex. Model II was adjusted for age, sex, hypertension, diabetes mellitus, cardiovascular disease and smoking history.

## 4 Discussion

A growing number of studies have shown an association between the TyG index and the TyG-BMI index and NAFLD. A cross-sectional study with 1727 participants revealed that the TyG-BMI was a reliable predictor of NAFLD and moderate to severe fibrosis in U.S. adults (14). Another prospective cohort study involving 772 participants reported that the TyG index had moderate predictive value for cardiovascular events in patients with NAFLD (15). In addition, a further study with 10,390 participants revealed that the TyG-BMI was significantly associated with all-cause mortality, cardiovascular-related mortality, and diabetes-related mortality among adults with NAFLD in the United States; a more pronounced nonlinear positive association was observed among participants without progressive fibrosis (16). Taken together, these studies revealed an association between the TyG index and the TyG-BMI index with NAFLD, but it is worth noting that most of these studies recruited the general population rather than specific high-risk groups.

Coronary artery disease (CAD), the most common cardiovascular disease and a leading cause of death worldwide, is often treated with percutaneous coronary intervention (PCI). A retrospective cohort study involving 1,353 participants revealed that the TyG index can be used as a prognostic indicator of cardiovascular events after PCI (17). Another retrospective study involving 1,158 patients who had previously undergone coronary artery bypass grafting for acute coronary syndrome revealed that a higher TyG index was independently associated with an increased risk of serious adverse cardiac events (MACCEs), with a progressively stronger predictive effect of MACCEs (18). An analysis of 1,438 participants revealed a proportional association between higher TyG-BMI and an increased incidence of MACCEs in older or female patients (19).

Given the association between NAFLD and increased CVD risk, and the predictive value of the TyG index for both NAFLD and CVD, few studies have examined the predictive power of the TyG and TyG-BMI indices for NAFLD in PCI patients. The present study aims to address this gap.

The TyG and TyG-BMI indices, as markers of insulin resistance (IR), are strongly associated with the development of CHD and PCI. While the exact mechanisms are not fully understood, these indices are linked to insulin resistance, systemic inflammation, oxidative stress, lipid metabolism disorders, visceral fat accumulation, and endoplasmic reticulum stress. The TyG and TyG-BMI indices not only reflect IR but also correlate with these mechanisms, explaining the high prevalence of NAFLD in PCI patients.

Insulin resistance (IR) is defined as a reduced response to physiological insulin levels. IR is a key driver of several modern diseases, including metabolic syndrome, NAFLD, atherosclerosis, and type 2 diabetes mellitus (T2DM), playing a crucial role in their progression. IR leads to impaired glucose metabolism and organ dysfunction, particularly in the liver and heart (20). IR impairs the PI3K-NO pathway and enhances the MAPK-ET-1 pathway, leading to endothelial dysfunction, atherosclerotic plaque formation, exacerbated inflammation, and increased thrombosis risk, thereby worsening cardiovascular disease progression (21, 22). In NAFLD patients, impaired liver function fails to suppress excess glucose and fatty acid production, leading to local lipid and glucose accumulation that initiates and worsens IR. This results in systemic metabolic disorders and vascular endothelial damage, explaining the high risk of coronary heart disease in NAFLD patients.

Systemic inflammation plays a key role in the development of CAD and NAFLD. Haukeland’s analysis of high-quality serum samples from 47 NAFLD patients found that mild systemic inflammation, involving CCL2 and MCP-1, is prevalent and plays a key role in the disease process (23). Lipid accumulation in hepatocytes triggers local inflammation and immune cell activation. Activated immune cells release proinflammatory cytokines and chemokines, causing systemic inflammation. Signals from adipose tissue also activate and recruit hepatic immune cells, promoting inflammation that leads to cell damage and death, accelerating NAFLD progression (24). Therefore, systemic inflammation-induced IR increases the risk of NAFLD in CAD patients.

Dyslipidemia plays an important role in the development of coronary heart disease and non-alcoholic fatty liver disease. Atherogenic dyslipidemia, manifested by elevated plasma triglycerides, increased numbers of small, dense low-density lipoprotein (sdLDL) particles, and reduced levels of HDL-C, is prevalent in patients with varying degrees of NAFLD. These dysregulated fat stores in the liver or other tissues are often accompanied by cardiometabolic dysfunction, which increases the risk of cardiovascular events (25).

Visceral fat accumulation plays a key role in the development of CAD and NAFLD. The accumulation of visceral fat is closely associated with the chronic release of free fatty acid (FFA) into the bloodstream, which can trigger insulin resistance in the liver. Excessively produced triglycerides, sdLDL and LDL particles tend to deposit in peripheral blood vessels, contributing to the development of atherogenic dyslipidemia (26). Studies have shown that abdominal obesity is an independent risk factor for arterial disease in the upper and lower extremities (27). In addition, elevated FFA levels can directly or indirectly activate gluconeogenesis in the liver and reduce liver insulin sensitivity. A decrease in insulin sensitivity leads to an increase in the production of very low-density lipoprotein (VLDL) and a reduction in HDL cholesterol, which further accelerates the progression of atherogenic dyslipidemias (28). The accumulation of ectopic fat and the subsequent release of metabolites as well as the activation of inflammatory pathways trigger a series of pathophysiological changes both locally and systemically, which ultimately lead to the development of NAFLD and CVD (29). Visceral adipose tissue acts as an autonomous endocrine organ that secretes various adipokines such as tumor necrosis factor-α (TNF-α), interleukin-6 (IL-6) and interleukin-10 (IL-10). These adipokines can not only trigger chronic inflammatory reactions, but also disrupt insulin signaling in liver cells, impair insulin sensitivity and thus promote the development of atherogenic dyslipidemia and the accumulation of free fatty acid (FFA) in liver cells (30, 31). Therefore, the accumulation of lipids in peripheral tissues and the liver leads to lipotoxic stress in mitochondria and the endoplasmic reticulum, which in turn leads to alterations in mitochondrial function and increased production of reactive oxygen species (ROS) (32). On the one hand, endoplasmic reticulum imbalance and oxidative stress have been shown to influence metabolic inflammatory responses by inducing endothelial dysfunction, increasing the susceptibility of patients with NAFLD to cardiovascular events (33); on the other hand, the resulting oxidative stress also promotes lipid peroxidation of polyunsaturated fatty acids, leading to the formation of toxic aldehyde products and causing lipid damage. These oxidatively modified lipids have an increased atherogenic effects (34).

Endoplasmic reticulum stress (ERS) plays a central role in the development of coronary artery disease (CHD) and NAFLD. ERS is profoundly pathophysiologically linked to NAFLD, CHD, and IR through the induction of apoptosis. In NAFLD, hepatocyte apoptosis exacerbates the inflammatory response and fibrosis in the liver (35). In addition, ERS disrupts insulin signaling and promotes the development of IR, which is not only a key factor in the progression of NAFLD but also an independent risk factor for CHD (36, 37). In CHD patients, cardiomyocytes undergo ERS-induced apoptosis, which further compromises myocardial function and structural integrity. Thus, ERS regulates a network of apoptotic and metabolic dysregulation that tightly links NAFLD, CHD, and IR, highlighting its central role in the development of these diseases and its potential as a therapeutic target.

In summary, IR, as a core pathophysiological process in patients with NAFLD, CHD, and those undergoing PCI (20, 38), contributes to the onset and progression of these conditions through a variety of mechanisms including systemic inflammation, oxidative stress, dyslipidemia, visceral obesity, and ERS. The TyG and TyG-BMI indices, as validated tools for assessing IR, not only reflect IR status, but also closely correlate with the aforementioned pathophysiological mechanisms, explaining the correlation between these indices and the high prevalence of NAFLD in PCI patients (5, 39, 40). Therefore, an in-depth understanding of the relationships between TyG and TyG-BMI indices and these diseases is essential for identifying at-risk individuals and optimizing preventive and therapeutic strategies.

Currently, conventional methods for assessing NAFLD risk in clinical practice have notable limitations: liver enzyme levels lack sufficient sensitivity; imaging modalities such as ultrasound and FibroScan require specialized equipment and entail relatively high costs; non-invasive fibrosis scores (e.g., FIB-4, NFS, APRI), although practical, were primarily developed for fibrosis risk assessment in the general population and their applicability and diagnostic performance in the specific context of PCI patients remain to be fully validated. Furthermore, liver biopsy, the gold standard for diagnosis, is invasive and thus unsuitable for large-scale screening. In contrast, the triglyceride-glucose (TyG) index and its derivative, TyG-BMI, offer significant clinical advantages. These indices are calculated from routinely available laboratory and anthropometric data—namely fasting triglycerides, glucose, and body mass index—making them highly feasible, cost-effective, and suitable for large-scale screening and longitudinal monitoring.

Patients undergoing percutaneous coronary intervention (PCI) are typically concerned about the risk of cardiac events, such as recurrent myocardial infarction or in-stent restenosis. However, this study highlights that this population is also at high risk for non-alcoholic fatty liver disease (NAFLD) and liver fibrosis. The TyG index is a reliable surrogate marker of insulin resistance, a central pathophysiological mechanism in the development and progression of NAFLD, thereby providing strong biological plausibility. The use of TyG-based indices could therefore facilitate large-scale screening and regular monitoring of liver disease in post-PCI patients, promoting a paradigm shift in clinical management from a “cardio-centric” approach to a more integrated “cardiometabolic and hepatic” care model.

There are several limitations to this study. First, this was a retrospective study with a limited sample size; the single-center nature and small sample size may have led to selection bias. Although a multivariable logistic regression was conducted to account for common confounding factors, the groups were not rigorously matched, and the differences between them were not adjusted for. Second, severe PCI patients requiring coronary vein bypass surgery or rheumatic heart disease were not included in this study, which may have led to an underestimation of the predictive power of the TyG and TyG-BMI indices for NAFLD. Third, the lack of liver enzyme data means that although ultrasonography is relatively accurate in detecting moderate to severe hepatic steatosis, it is less sensitive in detecting mild steatosis, which may lead to the underdiagnosis of mild NAFLD. Finally, the cross-sectional design of this study meant that although a positive correlation was found between the TyG and TyG-BMI indices and the prevalence of NAFLD in patients with PCI, no definitive conclusions could be drawn about their predictive value. Future large-scale, multicenter prospective studies are needed to further validate the predictive efficacy of the TyG and TyG-BMI indices for the risk of NAFLD in patients undergoing PCI.

## 5 Conclusions

In conclusion, this study demonstrated a significant association between the TyG index and the TyG-BMI and the risk of NAFLD in patients with PCI, and a positive correlation with the degree of liver fibrosis. As innovative markers of IR, the TyG index and TyG-BMI index have promising potential for wide application in primary health care facilities and community health services because of their simplicity, cost-effectiveness, and reliability. These results support the use of the TyG index and TyG-BMI index as valid tools for assessing the risk of NAFLD in patients undergoing PCI and provide a basis for follow-up studies. Future studies could validate these results with prospective cohort studies and explore additional biomarkers to improve their predictive accuracy.

## Data Availability

The raw data supporting the conclusions of this article will be made available by the authors, without undue reservation.
